# Development and application of a blubber-based detection protocol for phthalate ester metabolites in bottlenose dolphins (*Tursiops truncatus*)

**DOI:** 10.1007/s11356-026-38215-8

**Published:** 2026-09-11

**Authors:** Maggie A. Knight, Emily C. Pisarski, Randall S. Wells, Miranda K. Dziobak, Edward F. Wirth, Paul A. Sandifer, Amanda J. Moors, Leslie B. Hart

**Affiliations:** 1 Grice Marine Laboratory, College of Charleston, Charleston, SC USA; 2 NOAA National Centers for Coastal Ocean Science, Hollings Marine Laboratory, 331 Fort Johnson Rd, Charleston, SC USA; 3 Sarasota Dolphin Research Program, Brookfield Zoo Chicago, Mote Marine Laboratory, Sarasota, FL USA; 4 Department of Public Health Sciences and Administration, College of Charleston, Charleston, SC USA; 5 Center for Coastal Environmental and Human Health, College of Charleston, Charleston, SC USA; 6 Chemical Sciences Division, National Institute of Standards and Technology, Hollings Marine Laboratory, 331 Fort Johnson Road, Charleston, SC USA

**Keywords:** Endocrine disruptors, Environmental exposure, Marine mammals, Method development, High-performance liquid chromatography

## Abstract

Phthalates (PAEs) are high production volume plasticizing chemicals linked to endocrine disruption in mammals. Phthalates are released from the products to which they are added, leading to environmental exposure for humans and wildlife. Recent studies of bottlenose dolphin (*Tursiops truncatus*) urine have demonstrated prevalent exposure to phthalates. This study developed a protocol for phthalate ester metabolite (mPAE) detection in bottlenose dolphin blubber (~ 200 mg), a matrix that can be sampled remotely, and has been used to assess contaminant exposure (e.g., persistent organic pollutants and trace metals) in cetaceans in the past. Sonication in ammonium acetate, solid-phase extraction, and high-performance liquid chromatography-tandem mass spectrometry were used to extract and quantify mPAEs in blubber, and the finalized method was applied to samples (*n *= 27) collected during dolphin health assessments conducted in Sarasota Bay, FL, USA in 2016, 2017, 2022, and 2023. Detectable concentrations of three mPAEs (mono-2-ethylhexyl phthalate (MEHP; *n *= 6; censored mean: 3.0 ng/g wet weight; 5.2–12.4 ng/g ww), monoethyl phthalate (MEP; *n *= 2; ng/g ww; 39.7–50.1 ng/g ww), monoisodecyl phthalate (MIDP; *n *= 3; 26.0–27.4 ng/g ww)) were observed across the dataset, with at least one mPAE detected in 37% of sampled dolphins. Blubber provides a remote sampling option to investigate PAE exposure, particularly high-molecular-weight compounds, in populations where health assessments are not feasible. Blubber samples of the size analyzed in this study can be obtained via remote biopsy dart sampling, a method used globally to monitor contaminants in marine mammals. This approach facilitates exposure assessments in at-risk or remote populations, increases the efficiency of field sampling efforts, and reduces stress for animals.

**Supplementary Information:**

The online version contains supplementary material available at https://doi.org/10.1007/s11356-026-38215-8.

## Introduction

### Introduction to phthalates and their sources

Phthalates (PAEs) are a class of manufactured chemicals derived from phthalic acid (benzene-1,2-dicarboxylic acid) that are colorless with almost no odor (ATSDR 2008). Phthalates are commonly added to plastics to make them flexible (ATSDR 2008), but they are also added to personal care products (e.g., soap, shampoo, and conditioners), medications, pesticides, and cleaning products to enhance fragrance and durability (ATSDR 2008; Centers for Disease Control and Prevention 2017). Global phthalate production was estimated at 8 million metric tons per year in 2011 and is estimated to reach 10.5 million tons in 2026 (Ceresana 2019; Net et al. 2015). Today, frequently used phthalate compounds include diethyl phthalate (DEP), di-n-butyl phthalate (DBP), butyl benzyl phthalate (BBP), di(2-ethylhexyl) phthalate (DEHP), diisodecyl phthalate (DIDP), and diisononyl phthalate (DINP), which are common components of personal care products, nail polish, adhesives and sealants, polyvinyl chloride (PVC) plastics, rubber products, and automotive fuel products, respectively (ATSDR 2008; Koniecki et al. 2011; Staples et al. 1997; U.S. Environmental Protection Agency 2026).

Due to their widespread use, phthalates are present in every environmental compartment. Both phthalates and their metabolites (mPAEs) have been detected in seawater around the world (*reviewed in* Hidalgo-Serrano et al. 2022). Additionally, it has been reported that concentrations of these compounds vary seasonally, with higher concentrations in dry seasons compared to wet seasons, due to seawater dilution caused by increased river flow during rainy periods (Heo et al. 2020; Wang et al. 2021). Phthalates have been detected in sediments worldwide, with low-molecular-weight compounds most often found in coastal areas because of their use in personal care products (*reviewed in* Hidalgo-Serrano et al. 2022 and Paluselli and Kim 2020). Generally, PAE concentrations are higher in sediments than in surrounding seawater due to the lipophilic properties of PAEs, especially high-molecular-weight compounds, which enable them to absorb carbon-rich particulate matter (Wang et al. 2021; Zhang et al. 2020). Once phthalates enter the marine environment, they are distributed in the food web (Net et al. 2015) and taken up by and potentially concentrated in large fauna such as cetaceans (Baini et al. 2017; Fossi et al. 2014; Hart et al. 2018Rian al. 2020).

### Phthalate metabolism and preferred sampling matrix

Exposure to phthalates may occur via ingestion, inhalation, or dermal absorption (Centers for Disease Control and Prevention 2017; Net et al. 2015). Different phthalates have varying exposure routes, owing to their diverse applications in numerous products. In humans, dermal exposure is considered the major route of exposure to diethyl phthalate (DEP), which is the compound often found in personal care products such as soap, shampoo, and hair conditioners (Koniecki et al. 2011). In contrast, the main route of exposure to plasticizing phthalates such as DEHP is through inhalation or oral ingestion (Net et al. 2015). After uptake, phthalates are metabolized in a two-step process that includes hydrolysis and conjugation. In studies of rats, the majority of doses of both DEP (82–88%) and DEHP (42–96%) were eliminated within 24 h, no matter the administration method (Kluwe 1982). Hydrolysis of diester parent compounds to monoester metabolites occurs in the liver and small intestines (Kluwe 1982), and while this step detoxifies most xenobiotics, the monoester of DEHP, MEHP, is actually more bioactive than its parent compound (Ventrice et al. 2013). Conjugation occurs in several organs including the liver, lungs, small intestine, and kidney, forming hydrophilic glucuronide conjugates that are easily mobilized for excretion in urine (Silva et al. 2004; Ventrice et al. 2013).

To date, urine has been considered the most reliable matrix for phthalate exposure assessment in humans (Silva et al. 2005). This is because phthalates are made more hydrophilic and therefore more soluble in urine as they are metabolized (Frederiksen et al. 2007; Silva et al. 2004). In addition to urine, exposure assessments in humans have also used non-urine matrices such as breast milk, saliva, serum, and amniotic fluid (Bräuner et al. 2022; Frederiksen et al. 2007; Silva et al. 2005). Several phthalate metabolites have been detected at high proportions in breast milk samples tested in multiple cohort studies (Frederiksen et al. 2007).

### Phthalate exposure in marine fauna and study rationale

Marine mammals, particularly bottlenose dolphins (*Tursiops truncatus*), are widely considered ideal sentinels of marine ecosystem health. As long-lived, coastal, top-level predators with mammalian physiology similar to humans, they are vulnerable to many of the same environmental threats (Bossart 2011). Their protected status under the Marine Mammal Protection Act, coupled with their high public visibility as coastal species, has made them an important species for health monitoring and conservation efforts. The Sarasota Bay, FL, dolphin population has been studied continuously for over 50 years, making it one of the best-characterized marine mammal populations in the world (Wells et al. 2005). This extensive baseline allows for detailed interpretations of health trends and contaminant exposure over time, both within the population and in comparison to human health risks. An additional advantage to using marine mammals as ecosystem sentinels is their unique fat storage systems. Blubber is found in a variety of species including whales, seals, walruses, sea lions, and polar bears, and can act as a reservoir for anthropogenic contaminants (Bossart 2011). Blubber is a highly organized tissue composed of 80–90% lipids, and plays key roles in buoyancy, thermal insulation, and energy storage (Yu et al. 2022).

Its lipid-rich composition makes it particularly relevant for studying the accumulation of lipophilic environmental pollutants. The metabolic activity of blubber can vary by layer, age, body region, and environmental conditions (Montie et al. 2008; Yu et al. 2022), allowing researchers to potentially gain insight into both short- and long-term exposure depending on sampling depth and context.

Previous studies in Sarasota Bay have shown that approximately 75% of free-ranging dolphins tested had detectable levels of multiple phthalate compounds, as measured in urine (Dziobak et al. 2021; Hart et al. 2018). However, urine sampling requires catheterization during catch-and-release assessments, which is logistically demanding, expensive, and may be stressful to the animals. In contrast, blubber samples can be collected quickly and remotely from free-swimming dolphins using dart biopsy methods (Noren and Mocklin 2012), which are less invasive and have been used extensively across marine mammal contaminant studies (e.g., Baini et al. 2017; Fair et al. 2006; Wells et al. 2005).

Although studies have used blubber to conduct phthalate exposure assessment in marine mammals, most have relied on small sample sizes or measured diester parent compounds, which are prone to background contamination (Fossi et al. 2014, *n *= 5 fin whales; Routti et al. 2021*n *= 7, 6, 5 for blue whales, fin whales, and bowhead whales, respectively). Measuring monoester phthalate metabolites (mPAEs) instead, particularly those with higher molecular weights, may provide a more accurate indication of exposure, as these compounds are less likely to be artifacts from sampling or sample processing, and some may preferentially accumulate in lipid-rich tissues. Evidence from studies of human breast milk, which has both aqueous and lipid characteristics, supports the potential of lipid-rich matrices to carry and retain mPAEs (Frederiksen et al. 2007). Additionally, blubber may capture metabolites too hydrophobic to be efficiently metabolized for excretion in urine, potentially extending the window of detectable exposure.

The objectives of this study were two-fold: (1) develop a method for mPAE isolation from blubber to build upon previous methods that have quantified mPAEs in solid tissue matrices (Rian et al. 2020; Valton et al. 2014) and (2) apply the new protocol to measure phthalate metabolite concentrations in blubber samples collected from free-ranging bottlenose dolphins in Sarasota Bay, FL. Ultimately, this new method focusing on a new tissue matrix will allow for exposure assessment that can complement standard protocols that utilize urine, and potentially expand surveillance to areas where dolphins are particularly at risk of phthalate exposure and corresponding adverse health events (e.g., urban estuaries, developed coastlines) and where acquisition of urine samples may be impractical.

## Materials and methods

### Materials

Optima™ grade solvents (HPLC water, ethyl acetate, acetone, methanol) for cleaning, extraction, and mobile phases were obtained from VWR (Radnor, Pennsylvania). Formic acid (99.0+%, Optima™ LC/MS Grade), acetic acid, ammonium acetate, and sodium phosphate (ACS grade), and acetonitrile (99.0+%, Optima™ LC/MS Grade) were supplied by Fisher Scientific (Hampton, NH). Bond Elut NEXUS cartridges for solid-phase extraction (SPE), were obtained from Agilent Technologies (Santa Clara, California).

Twelve phthalate metabolite native standards, 4-methylumbelliferone (a reaction intermediate), and their isotopically labeled counterparts (^13^C_4_-monoethyl phthalate, ^13^C_4_-mono-2-ethylhexyl phthalate, ^13^C_4_-mono-(2-ethyl-5-oxohexyl)phthalate, ^13^C_4_-mono-(2-ethyl-5-hydroxyhexyl)phthalate, monoisodecyl phthalate-d_4_, monocarboxy isononyl phthalate-d_4_, monoisononyl phthalate-d_4_, mono-n-octyl phthalate-^13^C_2_, ^13^C_4_-monomethyl phthalate, ^13^C_4_-monobenzyl phthalate, ^13^C_4_-monobutyl phthalate, ^13^C_4_-monoisobutyl phthalate, and ^13^C_4_−4-methylumbelliferone) were purchased from Cambridge Isotope Laboratories (Tewksbury, MA). Spike and internal standard mixes were made up in methanol with the final concentrations of analytes detailed in SI Table 1. The spike mix was prepared by diluting National Institute of Standards and Technology (NIST) Standard Reference Material (SRM) 3060 Monoester Phthalates in Acetonitrile 1:25 in acetonitrile and adding mono(carboxy-isononyl) phthalate (MCNP), 4-methylumbelliferone (4-MeUMB), monoisononyl phthalate (MINP), and monoisodecyl phthalate (MIDP) to encompass the full suite of mPAEs intended for analysis. Isotopically labeled 4-methylumbelliferone was dissolved in acetone at a concentration of 1000 ng/mL. β-glucuronidase (*Escherichia coli* K12) and 4-methylumbelliferone β-D glucuronide were purchased from Sigma-Aldrich (St. Louis, MO). D_6_-Bisphenol A recovery standard was acquired from CDN Isotopes (Pointe-Claire, QC, Canada) and was prepared at a concentration of 5000 ng/mL in methanol.

### Protocol development

Methods were adapted from previous studies using solid tissues (liver from harbor porpoise, *Phocoena phocoena*, Rian et al. 2020; muscle and liver from roach, *Rutilus rutilus*, Valton et al. 2014; urine from bottlenose dolphin, Hart et al. 2018). Our protocol was developed and validated using a single 50-g full-depth blubber sample collected during necropsy via scalpel biopsy from a freshly dead bottlenose dolphin stranded near Charleston, SC (animal ID: SC1704, collected and frozen for storage on Jan 19, 2017).

Twelve mPAEs were selected for screening, including both low- and high-molecular-weight mPAEs (Table 1). Spike samples contained a known amount of mPAEs (SI Table 1). Three reagent spikes and 16 matrix spikes of varying mass were prepared and analyzed during the protocol development process to determine adequate blubber and mPAE masses required for data assessment. The utilization of reagent blanks in concert with reagent spikes allowed for the determination of instrumental response, recovery of analytes and identification of potential process-related contamination respectively. Subsequently, the use of matrix spikes allowed for the quantification of the influence the blubber had on the instrumental protocols set by the preceding sample types.

**Table 1:** Phthalate metabolites (mPAEs) selected for analysis, their abbreviations, and their molecular weights

Analyte	Abbreviation	Molecular weight
Monomethyl phthalate	MMP	180.16
Monoethyl phthalate	MEP	194.18
Monobutyl phthalate	MBP	222.24
Monoisobutyl phthalate	MiBP	222.24
Monobenzyl phthalate	MBzP	256.25
Mono-(2-ethylhexyl) phthalate	MEHP	278.34
Mono-n-octyl phthalate	MNOP	278.35
Mono(2-ethyl-5-oxohexyl)ester	MEOHP	292.33
Monoisononyl phthalate	MINP	292.40
Mono-(2-ethyl-5-hydroxyhexyl)phthalate	MEHHP	294.34
Monoisodecyl phthalate	MIDP	306.40
Mono(carboxy-isononyl) phthalate	MCNP	336.40

Quality assurance/quality control (QA/QC) samples were processed along with blubber samples, and calibration standards included reagent blanks (HPLC water), reagent spikes (HPLC water + spike solution (analytes ranged in mass from 9.5 to 605.4 ng per 100 µL aliquot depending on analyte)), and matrix spikes (blubber (0.2 g) + spike solution (9.5 to 605.4 ng per 100 µL aliquot depending on analyte)) were prepared as described below.

### Phthalate metabolite extraction and quantification protocol

**Fig. 1:**
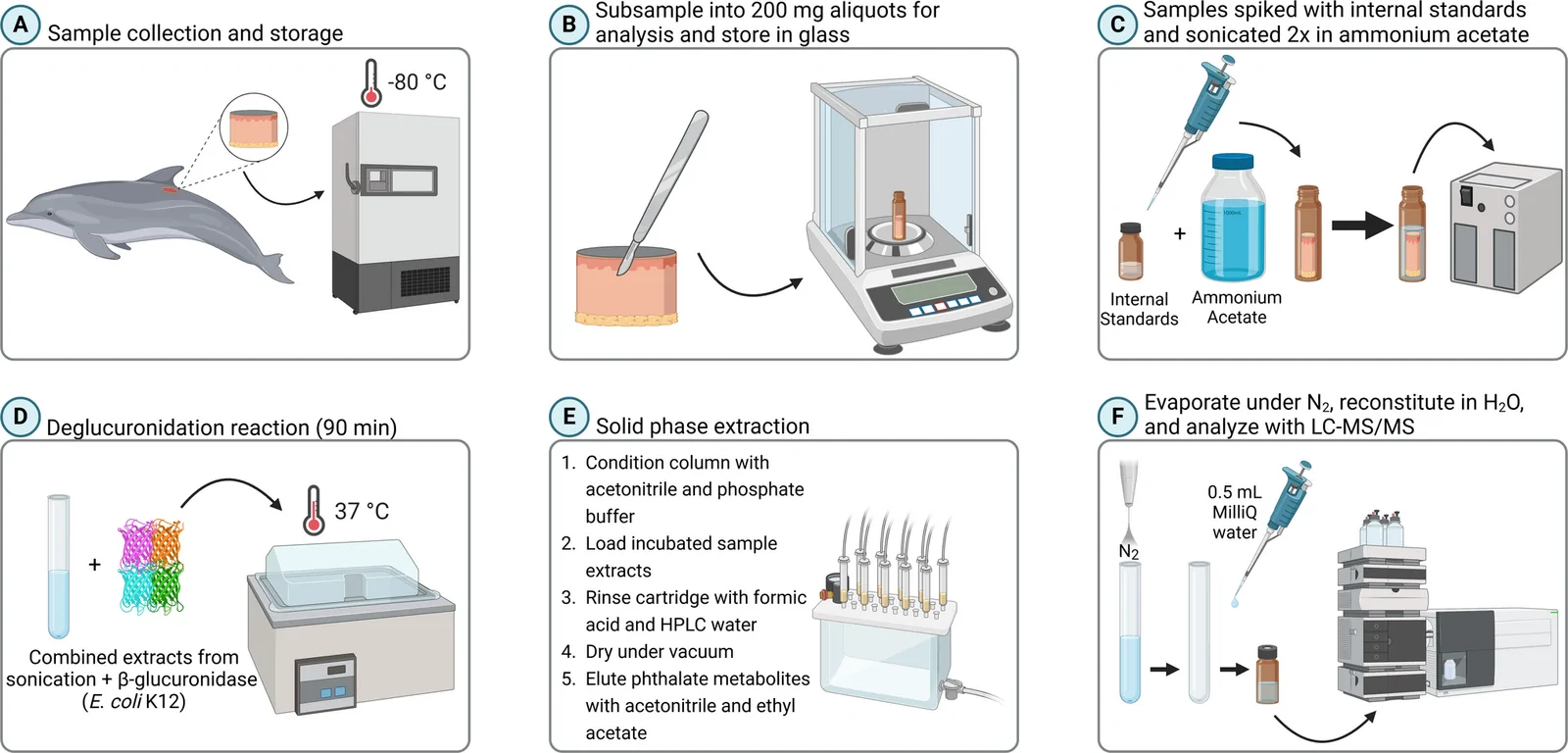
Workflow of finalized method for phthalate metabolite (mPAE) extraction from blubber. Sample collection and storage (**A**) was followed by sample sub-sectioning to reach the target sample extraction mass (**B**). At the time of extraction, samples were spiked with internal standards and sonicated (**C**), followed by an enzymatic deglucuronidation reaction (**D**). The samples were then processed with solid phase extraction (**E**), and then blown to dryness before reconstitution in water and analyzed with liquid chromatography-tandem mass spectrometry (LC-MS/MS) (**F**). Created in https://BioRender.com

Approximately 200 mg of blubber was placed into methanol-rinsed amber glass vials (Fig. 1B), submerged in 1 mL of 1 M ammonium acetate, and spiked with 100 µL isotopically labeled mPAE internal standards and 4-methylumbelliferone (SI Table 1). This mixture was sonicated twice for 20 min (Bransonic 2510 ultrasonic bath, VWR; Fig. 1C); after each round of sonication, 1 mL of the ammonium acetate mixture was pipetted out of the blubber vial and into a solvent-rinsed test tube. An additional 1 mL of ammonium acetate was added before the second round of sonication. After both rounds of sonication the extracts were combined. Twenty-five µL of 4-methylumbelliferone β-D glucuronide was then added to each combined sample and then vortexed. To complete deglucuronidation 250 µL aliquots of 1 M ammonium acetate and 5 µL of β-glucuronidase (*Escherichia coli* K12; 200 units/mL) were added to the samples and incubated at 37 °C for 90 min (Fig. 1D). The samples were then processed via solid-phase extraction using Bond Elut NEXUS cartridges (60 mg, 3 mL, Agilent) that were conditioned with a column volume each of acetonitrile and 0.14 M phosphate buffer (Fig. 1E) prior to sample loading. After loading the total combined volume of sample extract (approximately 2.5 mL), the cartridges were rinsed with 2 mL of 0.1 M formic acid and with 2 mL of HPLC water. Analytes were then eluted with one column volume of ethyl acetate followed by one column volume of acetonitrile. Following elution, samples were evaporated to dryness under nitrogen at 50 °C in a Biotage TurboVap LV Evaporator (Uppsala, Sweden) at 15 psi. Samples were then resuspended in 500 μL of HPLC water (water was the initial mobile phase for the LC-MS method). Finally, 10 µL of the isotopically labeled recovery standard was added, and samples were stored at −20 °C until analysis.

Isolation and quantification of each mPAE was conducted using high-performance liquid chromatography (HPLC; Agilent 1100; Phenomenex Gemini HPLC Column (3 um; 50 mm × 2 mm) analytical column) coupled to a triple quadrupole mass spectrometer (MS; Applied Biosystems Sciex API 4000) with electrospray ionization (ESI negative) interface (Fig. 1F). Separation was achieved over 11 min at a flow rate of 200–300 μL/min with a gradient mobile phase of HPLC-grade water with 0.1% acetic acid (A) and acetonitrile with 0.1% acetic acid (B). The mobile phase gradient was 1 min at 95%A:5%B, followed by a linear ramp to 10%A:90%B by 10 min, and column re-equilibration at 95%A:5%B. One ion transition was monitored for each analyte. Analyte sensitivity was optimized via manipulation of mass spectrometer parameters (e.g., delustering potential, entrance potential, collision energy, temperature, voltage) by direct infusion into the mass spectrometer. Retention times for each analyte were established by running individual analyte standards through the LC-MS method. Full method parameters are detailed in SI Table 8.

Sample integrations utilized Analyst software (version 1.6). Blubber samples were processed in batches of six along with QA/QC samples including reagent blanks, field blanks, reagent spikes, and matrix spikes for a total batch size of 10 (Dziobak et al. 2021; Hart et al. 2018). Reagent blank concentrations for each batch were subtracted from detected concentration values to account for any mPAE contamination resulting from laboratory processes. The calibration curve used in this study was extracted utilizing the method developed herein. The masses of analytes in each calibration point are shown in SI Table 7. The curve was composed of 10 points, with calibration point 7 corresponding to the masses of analyte present in the spike mix (SI Table 1). Calibration masses ranged from 0.1 to 4000 ng/mL, depending on the analyte and the calibration point. Calibration curves for each analyte were assessed for every batch and were rejected if the *r*^2^ correlation coefficient was less than 0.995. All calibration curves were calculated with linear regression. Calibration curves were run with every batch, ensuring that any retention time shifts for analytes were updated for instrumental conditions at the time of the run (SI Table 8).

### Method application to blubber samples from Sarasota Bay, FL, Bottlenose Dolphins

The Sarasota Bay dolphin community includes approximately 170 individuals with multi-decadal, multi-generational, year-round site fidelity to Sarasota Bay, FL (Fair et al. 2006; Wells et al. 2005). Long-term monitoring has included photo-identification studies, behavioral focal follow work, and periodic health assessments. Catch-and-release health assessments of inshore dolphins have been utilized to better understand endemic disease, establish baseline physiological measures, and evaluate exposure to, and potential effects of, chemical, biological, and physical stressors (Barratclough et al. 2019). During health assessments, free-ranging dolphins are encircled with a seine net (500 m by 4 m), temporarily restrained, and brought onboard a specialized platform for a veterinary examination (Barratclough et al. 2019; Fair et al. 2006). During this time, various biological samples (e.g., urine, blubber, gastric fluid, feces, blood) and measurements (e.g., morphological, ultrasonic, radiographic) are collected for health research studies, and then the animal is returned safely to the water.

Methods for blubber collection during health assessments have been reported previously by Wells et al. (2005). Briefly, wedge-shaped, full-thickness blubber samples (approximately 5 × 2.5 × 2 cm deep) were obtained by veterinarians with a scalpel and surgical forceps from a site below the caudal insertion of the dorsal fin (Fig. 1A). Before biopsy, the site was rinsed with water and cleaned, and lidocaine hydrochloride was administered for local anesthesia (Wells et al. 2005). Surgical instruments, foil, and sample receptacles were hexane-rinsed, acid-washed and autoclaved to minimize contamination. If samples were larger than 200 mg, they were subsampled to reach that mass in the lab while retaining full-thickness (Fig. 1B). Field blanks, prepared with DI water from NIST, were used for quality assurance/quality control (QA/QC) analysis, along with lidocaine and seawater from the capture site. These blanks, treated identically to samples, were stored at −80 °C in Teflon jars, and their values, if above limit of detection (LOD, “detectable concentrations”), were subtracted from detection concentration values to account for any mPAE contamination resulting from laboratory processes.

Reagent blank values, if detected, were also subtracted from detection concentration values to account for any mPAE contamination resulting from laboratory processes. Since extracted sample mass varied slightly across animals, the LOD differed for each mPAE in each sampled animal but was determined based on the lowest point of the calibration curve that could be detected on the instrument divided by the mass of blubber extracted (SI Table 6). In addition, continuing calibration verification standards were run with each batch of blubber samples to ensure the integrity of the calibration curve. Reagent spikes and matrix spikes used during method application had the same composition as during protocol development (SI Table 1).

### Statistical analysis

Descriptive statistics were used to summarize mPAE concentrations and the proportion of Sarasota Bay dolphins with concentrations above the LOD. For mPAE with greater than 20% of observations above LOD, means and standard deviations were calculated using Regression on Order Statistics (ROS) and Kaplan-Meier (K-M) methods (Helsel 2006). Comparisons by demography were not conducted due to limited sample sizes. For dolphins sampled more than once, the sample most recently obtained was used for analysis.

## Results

### Method development

Twelve phthalate metabolites (Table 1) were targeted for analysis. A series of blanks, reagent spikes, and matrix spikes was used to assess the repeatability of the method and separation, detection, and quantitation using LC-MSMS for the mPAEs. Additionally, retention times of all analytes established during method development were monitored continuously though the sample analysis process and the range of retention times from unknown batches are shown in SI Table 8. Percent recovery results for the 12 mPAEs measured in the fortified verification samples (matrix and reagent spikes) ranged from 31.4 to 133% (SI Table 2 and 4). MEHP recoveries in reagent spikes ranged from 31.4 to 112%, but averaged 60.6%; all other mean mPAEs recoveries were consistently between 80 and 120%. The average percent recovery for MEHP in matrix spikes was 90% (Table 2). Percent relative standard deviation was calculated for each analyte for all sample types through the method validation phase and during unknown sample analysis. The mean percent recovery in matrix spikes fell within ± 20% of 100 for all analytes. MEHP had the largest RSD in matrix (17.4) and reagent (73.8) spikes during the validation phase (Table 2), but these values aligned more closely with other analytes when field samples were analyzed.

**Table 2:** Average percent recovery values and associated relative standard deviations (RSD) for phthalate metabolite (mPAE) analytes in matrix and reagent spike samples for method development and final protocol extractions

Sample	Analyte											
	MBP	MBzP	MEHHP	MEHP	MEOHP	MEP	MIBP	MMP	MNOP	MCNP	MIDP	MINP
Validation matrix spike average (*n *= 16)	107	99.9	102	90.0	101	107	96.0	104	102	102	109	104
Validation matrix spike % RSD	11.2	4.36	8.89	17.4	5.97	10.7	8.92	11.5	4.25	7.01	10.7	4.09
Validation reagent spike average (*n *= 3)	108	97.7	102.3	60.6	101	95.6	96.6	99.6	82.4	100	92.3	95.5
Validation reagent spike % RSD	12.4	3.93	2.51	73.8	8.10	1.61	1.66	6.82	12.3	8.62	26.3	4.22
Field sample matrix spike average (*n *= 5)	101	99.7	107	72.0	114	102	87.9	104	102	95.2	85.2	93.4
Field sample matrix spike % RSD	2.45	4.73	8.57	28.1	8.81	3.72	6.19	6.57	5.82	3.69	4.98	12.0
Field sample reagent spike average (*n *= 5)	99.3	97.6	102	67.7	98.9	105	86.5	104	111	96.3	71.2	100
Field sample reagent spike % RSD	5.47	1.48	9.42	21.4	12.9	4.98	11.2	5.66	12.6	5.74	7.32	10.0

### Method validation

mPAEs were quantified in blubber from 27 dolphins opportunistically sampled in Sarasota Bay and the QA/QC samples from Sarasota Bay batches were compared to the QA/QC samples from the protocol development phase in Table 2. Matrix spikes for both protocol development samples and samples from Sarasota dolphins had higher percent recovery and lower RSD when averaged across all analytes. Solid-phase extraction (SPE) mean percent recoveries for method validation matrix spikes were > 90% for all analytes. All samples except for one (Matrix Validation 5; SI Table 2) fell within two *z*-scores of the mean. The mean percent recovery for QA/QC samples associated with unknown field samples fell within acceptable ranges for all 12 analytes (Table 2). In matrix spikes, 89.7% (*n *= 58) of analytes, and in reagent spikes, 82% (*n *= 53) of analytes fell within the acceptable recovery range (80–120%; Hennion 1999; Lipps, Braun-Howland, Baxter, eds 2023; U.S. Environmental Protection Agency 1995; see SI Table 2). However, it should be noted that percent recovery was low (60.6–90%), but consistent for MEHP for both matrix and reagent spikes. MIDP had a lower mean percent recovery across reagent spikes. Percent recoveries and RSDs for each analyte in the unknown samples were similar to their counterparts from method validation. Average LODs ranged from 2.2 ng/g ww (MEHP) to 27.2 ng/g ww (MCNP) (SI Table 6). The average LOD for MEP and MIDP were 18.5 ng/g ww and 2.5 ng/g ww, respectively (SI Table 6).

### Method application to Sarasota Bay bottlenose dolphin blubber

Blubber was collected from 27 dolphins sampled during catch-and-release health assessments conducted in 2016 (*n *= 9), 2017 (*n *= 8), 2022 (*n *= 4), and 2023 (*n *= 6). Samples were obtained from 13 males and 14 females, ranging in age from 2 to 34 years, and 67% of sampled dolphins were classified as sexually immature (based on sex hormone concentrations; Table 3).

**Table 3:** Bottlenose dolphin characteristics with associated concentrations (ng/g ww) of detected blubber phthalate metabolites (mPAEs)

Dolphin ID	Sampling year	Age at sampling	Sex	Sexual maturity	mPAE concentration (ng/g ww)		
					MEHP	MEP	MIDP
F178	2016	21	Male	Mature	--	--	--
F188	2016	20	Male	Mature	--	--	--
F209	2016	12	Female	Mature	--	--	--
F223	2016	15	Female	Mature	--	--	--
F255	2016	3	Female	Immature	--	39.7	--
FB33	2016	34	Female	Mature	--	50.1	--
F259	2016	3	Female	Immature	--	--	--
F292	2016	4	Male	Immature	--	--	--
F294	2016	3	Male	Immature	--	--	--
F151	2017	17	Female	Mature	--	--	--
F263	2017	2	Female	Immature	--	--	--
F267	2017	2	Female	Immature	--	--	--
F269	2017	4	Female	Immature	--	--	--
F271	2017	23	Female	Mature	--	--	--
F296	2017	4	Male	Immature	--	--	--
F298	2017	4	Male	Immature	--	--	--
F306	2017	4	Male	Immature	6.5	--	--
F293	2022	3	Female	Immature	5.3	--	--
F316	2022	5	Male	Immature	7.2	--	--
F320	2022	3	Male	Immature	12.4	--	--
F322	2022	7	Male	Immature	--	--	--
F295	2023	2	Female	Immature	5.2	--	27.4
F277	2023	11	Female	Mature	--	--	--
F297	2023	3	Female	Immature	--	--	--
F292	2023	11	Male	Mature	11.7	--	--
F324	2023	2	Male	Immature	--	--	26
F326	2023	4	Male	Immature	--	--	26.3

--≤LOD (see SI Table 6 for details); *MBP*, *MBzP*, *MEHHP*, *MEOHP*, *MIBP*, *MMP*, *MCNP*, and *MNOP* had no detectable concentrations in this sample set

Thirty-seven percent (*n *= 10) of SSB blubber samples had detectable concentrations (“detect”) of at least one mPAE (Table 4). Detectable concentrations were observed for three mPAEs: MEHP (*n *= 6); MEP (*n *= 2); and MIDP (*n *= 3). Detectable concentrations ranged from 5.2 to 12.4 ng/g ww for MEHP, 39.7 to 50.1 ng/g ww for MEP, and 26.0 to 27.4 ng/g ww for MIDP (Table 4). Only one dolphin (F295), a 2-year-old female, had detectable concentrations of more than one mPAE (MEHP and MIDP; Table 4). There were two detects in 2016, one detect in 2017, three detects in 2022, and five detects in 2023. The oldest dolphin (FB33) sampled, a 34-year-old female, had the highest concentration of any analyte (MEP; 50.1 ng/g, Table 4).

**Table 4:** Summary of detected concentrations for three phthalate metabolites (mPAE) in bottlenose dolphin blubber sampled in Sarasota Bay, FL (*n *= 27), 2016–2017, 2022, 2023

	MEHP	MEP	MIDP
# detects	6	2	3
% of total above max LOD^a^ (*n *= 27)	22.2	7.4	11.1
Mean (s.d.) ng/g	3.0 (3.1)^b^	-	-
Minimum detectable concentration (ng/g)	5.2	39.7	26.0
Maximum detectable concentration (ng/g)	12.4	50.1	27.4

^a^See limits of detection (SI Table 6)

^b^Calculated for all individuals including non-detects via ROS (Helsel 2006)

## Discussion

### Phthalate metabolite detection in bottlenose dolphin blubber

This method was based on, and expanded upon methods that extracted mPAEs from roach (fish) muscle tissue and harbor porpoise liver (Rian et al. 2020; Valton et al. 2014). We utilized a smaller sample size (~ 200 mg) than the muscle method (2 g), but larger than the liver method (~ 100 mg). Our method utilized two rounds of sonication in ammonium acetate to extract mPAEs from the solid matrix of blubber as in Rian et al. (2020). Like all of the previous methods, we utilized β-glucuronidase to ensure that only deconjugated compounds were quantified. Sonication was followed by solid-phase extraction as in the urine and liver studies. Muscle samples were extracted via liquid/liquid extraction and then samples were cleaned with solid-phase extraction (Valton et al. 2014). All studies concentrated extracts under a nitrogen stream before analysis on LC-MS/MS. This study detected and quantified 12 mPAEs. The muscle method quantified concentrations of seven monoesters, and the liver study quantified 17.

The low, but consistent, mean percent recoveries for MEHP could be attributed to non-ideal chemical or physical behaviors of analytical systems that can be difficult to detect until after experimentation has concluded. Analytes can be lost from the analytical process before, during, and after extraction from a matrix (Kumar et al. 2022). Prior to extraction, a portion of the MEHP may have been irreversibly bound to components of the matrix (proteins, salt complexation; Kumar et al. 2022). During extraction, MEHP may have experienced incomplete elution from the SPE cartridge, as documented in previous studies of mPAEs (Kumar et al. 2022). Post-extraction, some analytes may experience ion suppression, where interfering compounds may co-elute with analytes of interest (Hart et al. 2018; Millipore Sigma 2021). Additionally, as MEHP is a metabolite of one of the most widely used diester phthalates, DEHP, contamination with DEHP in laboratory or field sampling materials and sample blanks has been documented, leading to a lower percent recovery due to the subtraction of blank contamination concentrations after samples were extracted (Valton et al. 2014).

While the percent recoveries for MEHP spikes were lower than other analytes in this work, they are similar to prior studies that were foundational to this study. The percent recovery for MEHP in spikes was similar to MEHP recovery in a study performed with roach liver, plasma, and bile (65%), and to recoveries for MBP, and MiNP in a study performed with bottlenose dolphin urine (68% ± 20% and 64% ± 16%, respectively) and recoveries in this study were higher for all other analytes that were also examined in previous research (Hart et al. 2018; Valton et al. 2014). While the percent RSD was high for MEHP during the method development phase, further sample processing showed consistent MEHP recoveries (~ 70%; % RSD ~ 25%) and these data meet generally accepted verification and validation criteria. The United States Environmental Protection Agency typically validates extraction methods with recoveries in the range of 70 to 130% (Hennion 1999; U.S. Environmental Protection Agency 1995), and the American Public Health Association recommends percent recoveries of 70–130% for volatile organics (Lipps, Braun-Howland, Baxter, eds 2023). This was also the percent recovery range utilized for previous work using dolphin urine (Dziobak et al. 2021).

### Phthalate metabolites in Sarasota Bay dolphins

Three mPAEs of the 12 scanned were detected in the analyzed samples: MEP, MEHP, and MIDP. Detectable concentrations of MEHP and MEP were also observed in urine samples from Sarasota Bay dolphins (Dziobak et al. 2021; Hart et al. 2018). MIDP was newly included in our analytical suite for dolphin blubber samples, highlighting the value of expanding metabolite screening. Because previous urine-based studies (Dziobak et al. 2021; Hart et al. 2018) did not include MIDP, its detection here cannot be interpreted as a novel exposure. This result further supports the use of blubber for assessing phthalate exposure, especially with a larger analyte suite. Additionally, a broad range of detected concentrations was found in the studies shown below (Table 5). While some metabolites have been detected in excess of 1000 ng/g ww, most metabolites were detected in the range of 1 to 100 ng/g ww, similar to the range detected in this study. This may provide further evidence that the method utilized here may be appropriate for analysis of fatty tissues of species other than *T. truncatus* in the future.

**Table 5:** Detected concentrations of various phthalate metabolites (mPAE) in solid tissue matrices in various marine and aquatic fauna (ng/g)

Species	Matrix	*n*	MBP	MBzP	MEHHP	MEHP	MEOHP	MEP	MIBP	MMP	MNOP	MCNP	MIDP	MINP	Study
Bottlenose dolphin (*n *= 1)	Skin biopsy	1	780.1	*	--	1770	--	--	--	--	--	--	--	--	Baini et al. 2017
Fin whale (*n *= 5)	Blubber, muscle	5	--	--	--	176.67	--	--	--	--	--	--	--	--	Fossi et al. 2014
Fin whale (*n *= 3)	Skin biopsy	3	983.7	32.1	--	*	--	--	--	--	--	--	--	--	Baini et al. 2017
Striped dolphin (*n *= 2)	Skin biopsy	2	*	*	--	1720	--	--	--	--	--	--	--	--	Baini et al. 2017
Risso’s dolphin (*n *= 1)	Skin biopsy	1	*	*	--	463.7	--	--	--	--	--	--	--	--	Baini et al. 2017
Harbor porpoise (*n *= 100)	Liver	100	37.2	3.02	1.39	39.9	0.61	5.99	41.1	2.24	1.19	--	--	--	Rian et al. 2020
Polar bear (*n *= 13)	Adipose tissue	13	< 0.2–0.63	*	*	*	*	*	< 0.2–1.2	*	--	--	--	--	Routti et al. 2021
Indo-Pacific humpback dolphin (*n *= 74)	Liver	75	35.1	26.3	5.5	183.8	--	6	27	--	16.7	--	--	--	Xie et al. 2023
Loggerhead sea turtle (*n *= 79)	Liver	79	5.5	46.3	4.64	27.4	*	77.6	4.11	33.4	5.29	--	--	--	Sanjuan et al. 2023
European eel (*n *= 117)	Muscle	117	174	2	94	282	34	33	206	5.7	82	--	--	--	Fourgous et al. 2016
Roach (*n *= 4)	Liver	4	1496	19.7	163	237	7.1	263	1607	18.9	17.7	--	--	--	Valton et al. 2014
Basking shark (*n *= 6)	Muscle	6	--	--	--	84.1	--	--	--	--	--	--	--	--	Fossi et al. 2014

–Not measured

*Not detected

The detection range for MEHP in this study was similar to concentrations found in Sanjuan et al. (2023) and Valton et al. (2014), but lower than other studies of this metabolite (Table 5). This study detected three mPAEs, and a wide range of phthalate metabolites have been detected in marine mammals in several tissue matrices (Fossi et al. 2014; Rian et al. 2020; Routti et al. 2021; Xie et al. 2023). Other metabolites beyond the 12 assessed in this study have also been detected in marine animals but they are not included in Table 5. Other solid tissue matrices such as liver (Rian et al. 2020; Sanjuan et al. 2023; Xie et al. 2023) and muscle (Fossi et al. 2014; Fourgous et al. 2016) tend to show a larger number of phthalates than found in blubber in this study or skin in others, but these tissues cannot be obtained from live dolphins. Studies that utilized skin biopsies for phthalate metabolite assessment only found two of the metabolites surveyed for in this study. These results demonstrate that blubber biopsy has promise for detecting phthalate exposure in marine mammals.

Exposure to MEHP could be attributed to the widespread use of the parent compound, DEHP, in rigid plastics including food packaging, medical devices, and polyvinyl chloride (PVC) products. Globally, approximately 97% of DEHP is used in the production of PVC and is added to PVC in a proportion up to 30% (Agency for Toxic Substances and Disease Registry 2008). Plastic pipes make up about 54% of the pipes installed worldwide, and production for PVC makes up 62% of the demand for DEHP (Heim and Dietrich 2007). MEP is a metabolite of DEP, a phthalate added to a variety of personal care products (Koniecki et al. 2011). DEP is used as a solvent vehicle for fragrance and cosmetic ingredients, as well as alcohol denaturant. It is the most frequently used phthalate in personal care products and cosmetics (Koniecki et al. 2011). MIDP is a metabolite of DIDP, a high-molecular-weight phthalate primarily used in the manufacture of rigid industrial plastics, polymers, coatings, and PVC (U.S. Environmental Protection Agency 2026). Stringent government regulations in North America and Europe on the use of DEHP in consumer products have resulted in its substitution with other less toxic phthalates, notably DINP and DIDP (Net et al. 2015). The typical content of DIDP in some flexible PVC products can be between 25 and 50% (Silva et al. 2007).

### Comparisons between phthalate metabolite detection in blubber and urine

The proportion of dolphins with detectable concentrations (37%) was lower than previous studies that used urine as an assessment matrix (~ 75%; Dziobak et al. 2021). MMP, MBP, MiBP, MBzP, MNOP, MEOHP, MiNP, MEHHP, and MCNP were not detected in any of the blubber samples analyzed. This is not entirely surprising, given previous work. Other studies utilizing bottlenose dolphin urine as a detection matrix analyzed a smaller suite of eight mPAEs (Dziobak et al. 2021; Hart et al. 2018). MBP, MiNP, MiBP, MEHHP, and MBzP were not detected in any dolphin samples in the 2018 study, although the nondetection of MBP and MiNP may have been due to method limitations (Hart et al. 2018). In the 2021 study, MiBP was not detected, and the detection percentages of MMP, MEOHP, MEHHP, MBzP, MBP, and MiBP were much lower (1.96–7.84%) compared to MEP and MEHP (29.41–54.90%; Dziobak et al. 2021).

The lower detection frequency of phthalate metabolites in blubber (37%) compared to urine (75%), and the smaller number of metabolites detected in this study compared to past work may be due to differences in the chemical properties of the matrices (blubber and urine) and between low- and high-molecular-weight phthalates. Low-molecular-weight phthalates (with ester side chains of one to four carbons), such as DMP, DEP, DBP, and DIBP, are more hydrophilic and tend to partition into urine, muscle, and liver rather than lipid-rich tissues like blubber (Rian et al. 2020; Routti et al. 2021). In contrast, high-molecular-weight phthalates (with ester side chains of five or more carbons), including DEHP, DOP, and DINP, are more lipophilic. The octanol–water partition coefficient (KOW) estimates a chemical’s lipophilicity and is widely used to predict bioaccumulation in lipid-rich tissues (Gossett et al. 1983). Short-branched, low-molecular-weight phthalates like MEP and MMP have lower log KOW values (1.86 and 1.37, respectively) than higher-molecular-weight compounds such as mBzP, MEHP, and MIDP (3.07, 4.73, and 5.71, respectively; US Environmental Protection Agency Estimation Programs Interface Suite™ for Microsoft® Windows, v 4.11; Table 6; U.S. Environmental Protection Agency 2017).

**Table 6:** Estimated LogKOW (octanol-water partition coefficient) values for 12 phthalate metabolites included in analysis for this study. Estimations provided by U.S. Environmental Protection Agency Estimation Programs Interface Suite™ for Microsoft® Windows

Analyte	Molecular weight	CAS	SMILES	Log Kow (KOWWIN v1.69 estimate)
Monomethyl phthalate (MMP)	180.16	4376-18−5	COC(=O)C1=CC=CC=C1C(O)=O	1.37
Monoethyl phthalate (MEP)	194.18	2306-33−4	CCOC(=O)C1=CC=CC=C1C(O)=O	1.86
Mono(2-ethyl-5-oxohexyl) phthalate (MEOHP)	292.33	40321-98−0	CCC(CCC(C)=O)COC(=O)C1=CC=CC=C1C(O)=O	2.68
Monoisobutyl phthalate (MiBP)	222.24	30833-53−5	CC(C)COC(=O)C1=CC=CC=C1C(O)=O	2.77
Monobutyl phthalate (MBP)	222.24	131-70−4	CCCCOC(=O)C1=CC=CC=C1C(O)=O	2.84
Monobenzyl phthalate (MBzP)	256.25	2528-16−7	OC(=O)C1=CC=CC=C1C(=O)OCC1=CC=CC=C1	3.07
Mono-(2-ethyl-5-hydroxyhexyl) phthalate (MEHHP)	294.34	40321-99−1	CCC(CCC(C)O)COC(=O)C1=CC=CC=C1C(O)=O	3.19
Mono(carboxy-isononyl) phthalate (MCNP)	336.4	2042194-73−8	CC(C)CCCCCCOC(=O)C1=CC=CC=C1C(=O)OC(=O)O	4.28
Mono-(2-ethylhexyl) phthalate (MEHP)	278.34	4376-20−9	CCCCC(CC)COC(=O)C1=CC=CC=C1C(O)=O	4.73
Mono-n-octyl phthalate (MNOP)	278.348	5393-19−1	CCCCCCCCOC(=O)C1=CC=CC=C1C(O)=O	4.80
Monoisononyl phthalate (MINP)	292.4	106610-61−1	CC(C)CCCCCCOC(=O)C1=CC=CC=C1C(O)=O	5.22
Monoisodecyl phthalate (MIDP)	306.4	31047-64−0	CC(C)CCCCCCCOC(=O)C1=CC=CC=C1C(=O)O	5.71

This pattern could explain metabolic differences and subsequent differential partitioning of the two groups of phthalates. Low-molecular-weight phthalates are primarily excreted in urine as monoesters following initial hydrolysis (Silva et al. 2003). Parent compounds like DEHP, DiNP, and DiDP, which have higher log KOW values, may cause them to partition into blubber before metabolism. Hence, they have been previously detected in cetacean blubber (Andvik et al. 2024; Routti et al. 2021). Detection of high-molecular-weight monoester metabolites (e.g., MEHP, MIDP) in blubber may suggest some degree of partitioning into lipid-rich tissues after metabolism. In humans, phthalate metabolites found in breast milk are primarily unconjugated monoesters (Calafat et al. 2004; Main et al. 2006). Lower detection of phthalates in blubber may therefore result from the preferential excretion of low-molecular-weight metabolites in urine and other hydrophilic matrices, while only a small fraction partitions into blubber (Silva et al. 2003). It is unclear whether high-molecular-weight phthalates are substantially metabolized for urinary excretion, as previous dolphin studies did not include MNOP, MINP, MIDP, or MCNP (Dziobak et al. 2021; Hart et al. 2020). Furthermore, human exposure estimates based on urinary MEHP and MiNP levels may be underestimated, as these are not the primary excreted metabolites of DEHP and DiNP (Frederiksen et al. 2007).

Finally, the mPAEs detected in previous work may have not been detected here because of the variable and relatively high limits of detection in this study compared to studies that used urine as a sampling matrix. In previous studies that utilized urine, the LOD for MEHP ranged from 0.24 to 0.6 ng/mL for MEHP and 1 to 3.43 ng/mL (Dziobak et al. 2021; Hart et al. 2018). The LODs in this study were the same or greater than detected concentrations in previous work (Dziobak et al. 2021; Hart et al. 2018). The average LOD for MEHP and MIDP were 2.2 and 2.5 ng/g blubber, respectively. These were some of the lowest LODs across all mPAEs in this study, enabling detection of smaller amounts of these metabolites in analyzed samples. However, this trend does not hold true for MEP, for which the average LOD was 18.5 ng/g. It should be noted that MEP had the fewest detects (*n *= 2) of the three detected mPAEs and might have been more prevalent in this population had the average LOD been lower. The LODs were high in this study because a small mass of blubber (approximately 200 mg) was used for extraction. Sample sizes larger than 200 mg during method development tended to have excessive co-extracted material (e.g., lipids) that clogged SPE cartridges and made evaporation and concentration difficult. Reducing sample mass compared to prior work allowed for greater extraction capability, even if it did increase LODs.

### Limitations and strengths

This study faced several limitations related to both blubber protocol development and its subsequent application. First, protocol development relied on a single blubber sample from an adult coastal bottlenose dolphin from Charleston, SC. This individual was not from the Sarasota Bay population, and species, age, and sample location can impact blubber composition and the concentrations of contaminants within the tissue matrix (Montie et al. 2008; Ellisor et al. 2013) and may subsequently influence the extraction and recovery of analytes. Despite this, the developed method was successfully applied to blubber from Sarasota Bay bottlenose dolphins of varying age classes, demonstrating its broader utility. The relatively small sample mass (200 mg) used in the extraction protocol led to higher limits of detection (LOD) for some analytes, which may have impacted detection rates. Future work may want to investigate methods to mitigate lipid interference associated with larger sample masses. Additionally, the limited understanding of phthalate metabolism in blubber poses challenges in interpreting residue concentrations as measures of overall exposure. While studies in humans suggest age-related differences in phthalate metabolism (Domínguez-Romero and Scheringer 2019; Wittassek and Angerer 2008), it is unknown whether similar patterns exist in dolphins, highlighting a key area for future research. Finally, our metabolite panel was selected to enable comparisons with previous urine-based studies (Dziobak et al. 2021; Hart et al. 2018), which largely focused on low-molecular-weight phthalates. Future work should expand the analytical suite to include more high-molecular-weight mPAEs, which are more likely to partition into lipophilic tissues like blubber.

Despite the limitations, this study presents several important strengths that support the reliability and relevance of our findings. Most notably, our adaptation of existing methods to extract and quantify mPAEs from bottlenose dolphin blubber fills a critical gap in marine mammal health surveillance. To our knowledge, this is the first study to analyze phthalate metabolite concentrations in blubber samples collected from live, healthy dolphins. The success of this work was made possible by the long-term catch-and-release health assessments conducted in Sarasota Bay, which provided access to a diverse set of blubber samples. Building on established laboratory and analytical techniques (Dziobak et al. 2021; Hart et al. 2018; Rian et al. 2020; Valton et al. 2014), our method proved effective, with most analytes showing acceptable recovery values (70–130%; U.S. Environmental Protection Agency 1995). While MEHP recovery in reagent spikes was slightly below this range, its consistent performance in matrix spikes aligned with results from prior studies. Although the samples used in this study were not obtained via remote biopsy, our findings suggest that similar analyses may be feasible on remotely collected samples in future work. Remote biopsy typically yields full-thickness blubber samples ranging from 0.05 to 0.8 g (Noren and Mocklin 2012), comparable in size to the tissue used here. Incorporating remote biopsy could reduce labor and animal handling stress, increase sample sizes, and improve representation of wild populations. Challenges do remain. These include sample collection consistency, contamination risks, and the inability to collect paired matrices like urine during remote collection operations. However, the robustness and sensitivity of protocol reported herein demonstrate promise for future adaptation. Analytical precautions, such as using HPLC-grade reagents, eliminating plastic materials, and preparing fresh solvents, helped minimize contamination and ensure reliable extraction and quantification of mPAEs. Altogether, this study not only demonstrates a viable method for assessing phthalate exposure via blubber but also supports the potential for expanding its application to remote biopsy sampling in broader dolphin populations and in other marine mammals.

## Conclusions

This study adapted methods used to extract mPAEs from tissues of aquatic and marine fauna for use in bottlenose dolphin blubber. mPAEs have been well-studied in humans (Bräuner et al. 2022; Silva et al. 2007, 2005, 2004) and bottlenose dolphin urine (Dziobak et al. 2021; Hart et al. 2018). Phthalates have also been analyzed in marine mammal blubber, but with extremely small sample sizes or with samples exclusively taken from stranded animals. This advancement not only expands the repertoire of monitoring techniques but also offers a potentially less-invasive approach for ongoing surveillance beyond traditional health assessments. Furthermore, the rigorous precautions taken to mitigate contamination risks in the lab underscore the integrity of our data, bolstering confidence in the validity of our results. By standardizing methods for mPAE detection in bottlenose dolphin blubber, our study supports application of the approach of remote biopsy dart sampling, and facilitates the expansion of exposure surveillance to vulnerable areas, contributing to the understanding of associated adverse health effects and advancing marine conservation efforts.

This study also demonstrated that blubber is a viable matrix for assessing phthalate exposure in live dolphins. The successful adaptation of techniques from other tissues and species supports the potential for using remotely collected blubber biopsies in future research. These findings provide a foundation for further work into mPAE exposure and effects in marine mammals and emphasize the importance of continued method development and environmental monitoring in support of marine conservation.

Blubber is less effective than urine for detecting low-molecular-weight compounds, which are commonly found in personal care products, whereas high-molecular-weight compounds are primarily used in hard plastics (Hart et al. 2020). If future research aims to examine the trophic transfer of mPAEs from microplastics to dolphin prey and ultimately to dolphins (Merrill et al. 2023; Dool and Bosker 2022), focusing on high-molecular-weight compounds may be more informative. While blubber is a less reliable indicator of overall phthalate exposure compared to urine, it can still complement urine analysis, particularly for high-molecular-weight phthalate metabolites.

## Supplementary Information

Below is the link to the electronic supplementary material.

ESM 1 (PDF 547 KB)

## Data Availability

The authors declare that the data supporting the findings of this study are available within the paper and its supplementary information files. Copies of the data can also be obtained from the National Centers for Environmental Information: https://www.ncei.noaa.gov/access/metadata/landing-page/bin/iso?id=gov.noaa.nodc:0316133.

## Declarations

### NOAA disclaimer

This report has been reviewed and approved for publication according to NOAA’s Scientific Integrity Policy and Fundamental Research Communications (FRC) framework, and the National Ocean Service (NOS) process for FRC review. The opinions, findings, conclusions, and recommendations expressed in this report are those of the authors, and they do not necessarily reflect those of NOAA. Any use of trade, firm, or product names is for descriptive purposes only and does not imply endorsement by the US government. Mention of trade names or commercial products does not constitute endorsement or recommendation for their use by the US government.

### NIST disclaimer

Identification of certain commercial equipment, instruments, software, or materials does not imply recommendation or endorsement by the National Institute of Standards and Technology, nor does it imply that the products identified are necessarily the best available for the purpose.
