# Supplementary material for: Development and application of a blubber-based detection protocol for phthalate ester metabolites in bottlenose dolphins (Tursiops truncatus)
Source: Environ Sci Pollut Res Int. 2026 Sep 11;33(30):15553–67. doi: 10.1007/s11356-026-38215-8 (PMC13633138; doi:10.1007/s11356-026-38215-8)
Supplement: Supplementary file 1 [file 11356_2026_38215_MOESM1_ESM.pdf]

Detecting and Quantifying Phthalate Exposure Using Blubber from Bottlenose Dolphins (*Tursiops truncatus*)

Environmental Science and Pollution Research

Authors: Maggie A. Knight[1,2], Emily C. Pisarski[3], Randall S. Wells[4], Miranda K. Dziobak[5,6], Edward F. Wirth[3], Paul Sandifer[7], Amanda J. Moors[8], Leslie B. Hart[6]

Affiliations:
[1]Graduate Program in Marine Biology, Grice Marine Laboratory, College of Charleston, Charleston, SC
[2]Oak Ridge Institute for Science and Education (ORISE), Hollings Marine Laboratory, 331 Fort Johnson Road, Charleston, SC 29412, USA
[3]NOAA National Centers for Coastal Ocean Science, 331 Fort Johnson Rd., Charleston, SC, 29412, USA
4Sarasota Dolphin Research Program, Brookfield Zoo Chicago, c/o Mote Marine Laboratory, Sarasota, FL, 34236, USA
[5]Environmental Health Sciences, Graduate Program, University of South Carolina, Columbia, SC, USA.
[6]Department of Health and Human Performance, College of Charleston, Charleston, SC, USA.
[7]Center for Coastal Environmental and Human Health, College of Charleston, Charleston, SC, USA
[8] National Institute of Standards and Technology, Chemical Sciences Division, Hollings Marine Laboratory, 331 Fort Johnson Road, Charleston, SC 29412, USA

Corresponding author: Maggie Knight; knightma@g.cofc.edu; margaret.knight@noaa.gov

Supplementary Information

Supplementary Information Table 1. Masses of phthalate metabolite (mPAE) analytes present in 100 uL of spike mix

Analyte	Abbreviation	100 ul spike (ng)	100 uL internal standard (ng)
4-methylumbelliferone	4-MeUMB	399.6	100
Monobutyl phthalate	mBP	102.8	100
Monobenzyl phthalate	mBzP	48.1	100
Mono(2-ethylhexyl) phthalate	MEHP	9.5	100
Mono(2-ethyl-5-oxohexyl) phthalate	MEOHP	41.8	100
Monoethyl phthalate	MEP	411.9	100
Monoisobutyl phthalate	MIBP	55.7	100
Monomethyl phthalate	MMP	10.4	100
Mono(2-ethyl-5-hydroxyhexyl) phthalate	MEHHP	54.1	100
Monooctyl phthalate	MNOP	11.3	100
mono(carboxy-isononyl) phthalate	MCNP	605.4	100
Monoisononyl phthalate	MINP	390.5	100
monoisodecyl phthalate	MIDP	400.0	100

Supplementary Information Table 2: Matrix spike percent recoveries for method validation samples

	Analyte												
Sample	4-MeU MB	MBP	MBzP	MEHHP	MEHP	MEOHP	MEP	MIBP	MMP	MNOP	MCNP	MIDP	MINP
Matrix Validation 1	104	94.7	90.3	94.4	89.7	96.9	89.7	85.4	85.7	95.2	102	100	104
Matrix Validation 2	107	100	93.4	95.5	106	104	95.1	90	104	103	107	102	103
Matrix Validation 3	104	104	96	99.7	106	102	96.2	99.4	89.7	98.8	99.4	98.5	103
Matrix Validation 4	105	102	99.2	110	99.2	103	116	99	120	96.7	96.8	102	102
Matrix Validation 5	119	93.3	97.5	97.6	53.5	83.6	118	86.9	87.7	98.5	87.8	99.9	96.3
Matrix Validation 6	110	95.8	105	96.3	80.9	94.4	116	80.9	115	105	93.6	110	99.3
Matrix Validation 7	107	103	103	106	82.9	106	119	106	127	108	101	111	104
Matrix Validation 8	108	104	100	117	93.2	95.9	117	104	101	104	98.2	97	109
Matrix Validation 9	100	97.3	102	111	70.6	105	127	91.3	109	109	99.7	97.9	105
Matrix Validation 10	97.6	92.6	99.9	114	76.8	106	109	91.6	113	107	101	107	104
Matrix Validation 11	104	109	107	114	89.4	101	119	110	104	99.1	98	108	112
Matrix Validation 12	105	123	100	98.2	80	107	101	94.7	115	103	103	105	99.6
Matrix Validation 13	114	125	100	83.1	91	102	99.1	103	98.2	107	115	128	107
Matrix Validation 14	109	117	100	96.7	100	106	93.1	91.1	98.2	103	109	119	102
Matrix Validation 15	112	125	106	105	113	102	101	108	92.9	97.4	115	133	108
Matrix Validation 16	109	123	98.7	99.6	108	105	101	95.1	110	99.7	105	128	111

Supplementary Information Table 3: Matrix spike percent recoveries for Sarasota Bay dolphin batches

	Analyte												
Sample	4-MeUMB	MBP	MBzP	MEHHP	MEHP	MEOHP	MEP	MIBP	MMP	MNOP	MCNP	MIDP	MINP
Matrix Spike Batch 1	107	100	103	111	55.3	107	102	96.2	104	101	99.6	86	91.1
Matrix Spike Batch 2	103	105	95.0	92.0	59.7	110	108	81.1	101	111	98.1	89.3	97.5
Matrix Spike Batch 3	107	99.2	106	109	57.0	104	98.6	88.5	94.3	95.6	93.7	87.8	75.1
Matrix Spike Batch 4	93	99.2	96.9	107	94.5	121	98.4	86.9	106.8	104	93.5	84.9	99.6
Matrix Spike Batch 5	102	102	97.3	117	93.8	128	103	86.7	113	97.35	91.2	78.3	104

Supplementary Information Table 4: Reagent spike percent recoveries for method validation samples

	Analyte												
Sample	4-MeUMB	MBP	MBzP	MEHHP	MEHP	MEOHP	MEP	MIBP	MMP	MNOP	MCNP	MIDP	MINP
Reagent Validation 1	104	104	102	105	38.3	92	96.6	98.2	98	77.5	93.7	75.1	94.1
Reagent Validation 2	102	97.2	94.7	99.9	31.4	103	93.8	96.5	93.7	75.7	110	81.7	92.3
Reagent Validation 3	103	123	96.3	102	112	108	96.3	95	107	94.1	96.9	120	100

Supplementary Information Table 5: Reagent spike percent recoveries for Sarasota Bay dolphin batches

	Analyte												
	4-MeUMB	MBP	MBzP	MEHHP	MEHP	MEOHP	MEP	MIBP	MMP	MNOP	MCNP	MIDP	MINP
Reagent Spike Batch 1	109.75	94.65	95.43	115	62.63	86.36	101.26	85.93	104.85	125.66	101.26	73.25	100.81
Reagent Spike Batch 2	105.23	107.98	98.54	89.07	57.47	96.65	113.89	84.17	107.77	125.66	103.24	72.5	103.43
Reagent Spike Batch 3	106.75	96.21	97.09	99.83	58.53	89	103.69	102.7	93.59	108.85	93.82	78	85.59
Reagent Spike Batch 4	99.25	96.5	99.17	99.44	92.74	104.78	102.72	82.18	107.77	96.46	92.17	67.34	98.08
Reagent Spike Batch 5	103	101.17	97.71	106.67	67.05	117.7	101.99	77.27	104.85	99.12	91.18	64.75	113.44

Supplementary Information Table 6: Limits of detection (LOD) values (ng/g) for phthalate metabolites measured in bottlenose dolphin blubber samples.

ID	Mass	Year	MBP	MBzP	MEHHP	MEHP	MEOHP	MEP	MiBP	MMP	MCNP	MIDP	MINP	MNOP
FB33	0.3	2016	4	4.7	5.2	1.9	4.1	16	5.4	2	23.5	2.2	15.5	15.1
F178	0.2	2016	4.7	5.5	6.2	2.2	4.8	19	6.4	2.4	27.9	2.6	18.4	18
F188	0.2	2016	4.9	5.7	6.4	2.3	5	19.5	6.6	2.5	28.6	2.6	18.9	18.4
F209	0.2	2016	5.9	6.9	7.8	2.8	6	23.7	8	3	34.8	3.2	23	22.4
F223	0.2	2016	4.9	5.7	6.4	2.3	5	19.5	6.6	2.5	28.7	2.7	19	18.5
F255	0.2	2016	5.3	6.1	6.9	2.4	5.4	21	7.1	2.7	30.8	2.9	20.4	19.9
F259	0.2	2016	5.4	6.3	7.1	2.5	5.5	21.7	7.3	2.7	31.8	2.9	21.1	20.5
F292	0.2	2016	4.3	5	5.6	2	4.4	17.2	5.8	2.2	25.3	2.3	16.7	16.3
F294	0.2	2016	5.1	5.9	6.7	2.4	5.2	20.3	6.9	2.6	29.9	2.8	19.7	19.2
F267	0.2	2017	5.1	5.9	6.6	2.4	5.1	20.2	6.8	2.5	29.7	2.7	19.6	19.1
F151	0.2	2017	4.7	5.4	6.1	2.2	4.8	18.7	6.3	2.4	27.4	2.5	18.1	17.7
F306	0.2	2017	4.6	5.4	6.1	2.2	4.7	18.5	6.2	2.3	27.1	2.5	17.9	17.5
F269	0.2	2017	4.4	5.1	5.7	2	4.4	17.4	5.9	2.2	25.6	2.4	16.9	16.5
F263	0.2	2017	4.4	5.2	5.8	2.1	4.5	17.7	6	2.2	26	2.4	17.2	16.8
F296	0.3	2017	3.7	4.3	4.8	1.7	3.7	14.7	5	1.9	21.6	2	14.3	13.9
F271	0.2	2017	5	5.8	6.5	2.3	5.1	19.9	6.7	2.5	29.2	2.7	19.3	18.8
F298	0.3	2017	3.5	4.1	4.6	1.6	3.6	14	4.7	1.8	20.6	1.9	13.6	13.3
F293	0.3	2022	4	4.6	5.2	1.8	4	15.8	5.3	2	23.3	2.2	15.4	15
F316	0.2	2022	4.6	5.3	6	2.1	4.7	18.3	6.2	2.3	26.9	2.5	17.8	17.4
F320	0.3	2022	4.1	4.8	5.4	1.9	4.2	16.4	5.5	2.1	24.1	2.2	15.9	15.5
F322	0.2	2022	4.6	5.4	6.1	2.2	4.7	18.6	6.3	2.3	27.3	2.5	18	17.6
F295	0.2	2023	4.3	5	5.7	2	4.4	17.3	5.8	2.2	25.5	2.4	16.8	16.4
F297	0.3	2023	4	4.6	5.2	1.8	4	15.9	5.4	2	23.3	2.2	15.4	15
F292	0.2	2023	4.3	5	5.6	2	4.4	17.1	5.8	2.2	25.1	2.3	16.6	16.2
F277	0.2	2023	4.6	5.4	6	2.1	4.7	18.4	6.2	2.3	27	2.5	17.9	17.4
F324	0.2	2023	5.5	6.4	7.2	2.6	5.6	21.9	7.4	2.8	32.2	3	21.3	20.7
F326	0.2	2023	5.2	6	6.8	2.4	5.3	20.8	7	2.6	30.5	2.8	20.2	19.7
**Min**			3.5	4.1	4.6	1.6	3.6	14	4.7	1.8	20.6	1.9	13.6	13.3
**Max**			5.9	6.9	7.8	2.8	6	23.7	8	3	34.8	3.2	23	22.4
**Avg LOD**			4.6	5.4	6.1	2.2	4.7	18.5	6.2	2.3	27.2	2.5	18.0	17.5

Supplementary Information Table 7: Phthalate metabolite analyte masses (ng/mL) in the 10-point calibration curve utilized for this study

Analyte	Cal 1	Cal 2	Cal 3	Cal 4	Cal 5	Cal 6	Cal 7	Cal 8	Cal 9	Cal 10
mBP	1.03	2.57	5.14	10.28	25.70	51.40	102.81	257.02	514.04	1028.09
mBzP	0.48	1.20	2.41	4.81	12.03	24.05	48.10	120.26	240.52	481.03
MEHP	0.10	0.24	0.48	0.95	2.38	4.76	9.53	23.82	47.63	95.26
MEOHP	0.42	1.05	2.09	4.18	10.45	20.91	41.82	104.54	209.08	418.15
MEP	4.12	10.30	20.59	41.19	102.97	205.93	411.86	1029.66	2059.32	4118.64
MIBP	0.56	1.39	2.78	5.56	13.91	27.82	55.65	139.12	278.24	556.49
MMP	0.10	0.26	0.52	1.04	2.59	5.19	10.38	25.94	51.88	103.75
MEHHP	0.54	1.35	2.70	5.41	13.52	27.04	54.08	135.19	270.38	540.77
MNOP	0.11	0.28	0.56	1.13	2.82	5.64	11.29	28.22	56.43	112.87
MCNP	6.05	15.13	30.27	60.54	151.34	302.68	605.36	1513.40	3026.80	6053.60
4-MeUMB	4.00	9.99	19.98	39.96	99.90	199.80	399.60	999.00	1998.00	3996.00
MINP	3.90	9.76	19.52	39.05	97.62	195.24	390.48	976.20	1952.40	3904.80
MIDP	4	10	20	40	100	200	400	1000	2000	4000
d6 BPA (RS)	50	50	50	50	50	50	50	50	50	50

Supplementary Information Table 8: Mass to charge ratios, retention times, and retention time ranges in unknown samples for 12 phthalate metabolites selected for analysis in this study.

Native Analyte	Q1	Q3	RT (min)	RT range (min)	Declustering Potential (V)	Entrance Potential (V)	Collision Energy (eV)	Collision Cell Exit Potential (V)	R^2 range
mono-methyl phthalate	179	77	2.9	3.10-3.23	-37	-10	-27	-15	0.9979-0.9999
monoethyl phthalate	193.1	77	4.6	4.20-4.89	-28	-10	-22	-15	0.9983-1.0000
4-methylumbelliferone sodium salt	175	133	3.9	4.09-4.27	-37	-10	-29	-15	0.9993-1.0000
monobutyl phthalate	221	77	10.4	8.70-8.81	-33	-10	-29	-15	.9975-1.0000
monoisobutyl phthalate	221	77	10.1	8.56-8.68	-22	-10	-24	-15	0.9990-1.000
monobenzyl phthalate	255	183	11.1	9.22-9.38	-24	-10	-15	-15	0.9991-1.000
Mono-(2-ethylhexyl) phthalate	227	134	15	12.80-12.90	-22	-10	-20	-15	0.9952-0.9997
mono(2-ethyl-5-oxohexyl)ester	291	121	10.4	8.67-8.88	-22	-10	-20	-15	0.9994-0.9999
mono-(2-ethyl-5-hydroxyhexyl)phthalate	293	145	10.3	8.36-8.46	-40	-10	-22	-15	0.9971-0.9998
mono(carboxy-isononyl) phthalate	335.3	187	11.9	9.93-10.00	-32	-10	-20	-15	0.9991-0.9999
Monoisononyl phthalate	291.3	141	15.8	13.70-14.20	-33	-10	-29	-15	0.9919-1.0000
mono-n-octyl phthalate	276.9	126.9	15	13.00-13.40	-22	-10	-24	-15	0.9942-0.9999
monoisodecyl phthalate	304.8	154.9	15	14.50-14.70	-24	-10	-15	-15	0.9641-1.000
**INTERNAL STANDARD**									
$^{13}C_4$-monomethylphthalate	183	79	2.9	3.07-3.23	-37	-10	-27	-15	--
$^{13}C_4$-monoethyl phthalate	197	79	4.4	4.79-5.01	-28	-10	-22	-15	--
$^{13}C_4$-4-methylumbelliferone	179	135	3.9	4.08-4.26	-37	-10	-29	-15	--
$^{13}C_4$-mono-n-butyl phthalate	225	79	10.4	8.68-8.80	-33	-10	-29	-15	--
$^{13}C_4$-monobenzyl phthalate	259	186	11.1	9.26-9.41	-24	-10	-15	-15	--
$^{13}C_4$-mono-2-ethylhexyl phthalate	281	137	15	12.80-12.90	-22	-10	-20	-15	--
$^{13}C_4$-mono-(2-ethyl-5-oxohexyl)phthalate	297	124	10.4	8.36-8.44	-40	-10	-22	-15	--
$^{13}C_4$-mono-(2-ethyl-5-hydroxyhexyl)phthalate	297	145	10.3	8.37-8.45	-32	-10	-20	-15	--
Monoisodecyl Phthalate-d_4	309.2	154.9	16.8	14.90-15.00	-37	-10	-27	-15	--

Monocarboxy Isononyl Phthalate-d_4	339.3	187	11.6	9.92-10.00	-28	-10	-22	-15	--
Monoisononyl phthalate-d_4	295.4	141	15.8	13.90-14.10	-37	-10	-29	-15	--
mono-n-octyl phthalate - $^{13}C_2$	281.2	79	15	13.20-13.30	-33	-10	-29	-15	--

Supplementary Information Table 9. Z-scores for 12 phthalate metabolites and one reaction intermediate across 16 matrix validation spike samples, three reagent validation spike samples, matrix spikes in five unknown batches batches, and reagent spikes in five unknown batches. Each cell represents the z-score of a metabolite within a given validation or spike sample.

Sample	4-MeUMB	MBP	MBzP	MEHHP	MEHP	MEOHP	MEP	MIBP	MMP	MNOP	MCNP	MIDP	MINP
Matrix Validation 1	-0.61	-1.01	-2.20	-0.88	-0.02	-0.72	-1.53	-1.24	-1.55	-1.60	0.00	-0.78	-0.08
Matrix Validation 2	-0.03	-0.57	-1.49	-0.76	1.02	0.46	-1.06	-0.70	-0.03	0.20	0.70	-0.61	-0.31
Matrix Validation 3	-0.61	-0.23	-0.89	-0.29	1.02	0.13	-0.96	0.39	-1.22	-0.77	-0.36	-0.91	-0.31
Matrix Validation 4	-0.41	-0.40	-0.15	0.84	0.59	0.29	0.75	0.35	1.30	-1.26	-0.72	-0.61	-0.54
Matrix Validation 5	2.27	-1.13	-0.54	-0.53	-2.33	-2.92	0.93	-1.07	-1.39	-0.84	-1.98	-0.79	-1.88
Matrix Validation 6	0.54	-0.92	1.18	-0.67	-0.58	-1.13	0.75	-1.77	0.88	0.66	-1.17	0.07	-1.18
Matrix Validation 7	-0.03	-0.32	0.72	0.40	-0.45	0.79	1.01	1.16	1.88	1.35	-0.14	0.16	-0.08
Matrix Validation 8	0.16	-0.23	0.03	1.61	0.20	-0.88	0.84	0.93	-0.28	0.43	-0.53	-1.04	1.10
Matrix Validation 9	-1.37	-0.80	0.49	0.95	-1.24	0.62	1.71	-0.55	0.38	1.58	-0.32	-0.96	0.16
Matrix Validation 10	-1.83	-1.19	0.01	1.28	-0.84	0.79	0.15	-0.52	0.71	1.12	-0.14	-0.18	-0.08
Matrix Validation 11	-0.61	0.18	1.63	1.28	-0.04	-0.04	1.01	1.63	-0.03	-0.70	-0.56	-0.10	1.80
Matrix Validation 12	-0.41	1.36	0.03	-0.46	-0.64	0.95	-0.55	-0.15	0.88	0.20	0.14	-0.35	-1.11
Matrix Validation 13	1.31	1.53	0.03	-2.12	0.06	0.13	-0.71	0.81	-0.52	1.12	1.82	1.61	0.63
Matrix Validation 14	0.35	0.86	0.03	-0.62	0.64	0.79	-1.23	-0.57	-0.52	0.20	0.98	0.84	-0.54
Matrix Validation 15	0.93	1.53	1.41	0.29	1.47	0.13	-0.55	1.40	-0.96	-1.09	1.82	2.03	0.86
Matrix Validation 16	0.35	1.36	-0.27	-0.31	1.15	0.62	-0.55	-0.11	0.47	-0.56	0.42	1.61	1.56
Reagent Validation 1	1.00	-0.30	1.13	1.05	-0.50	-1.10	0.67	1.02	-0.23	-0.49	-0.75	-0.71	-0.34
Reagent Validation 2	-1.00	-0.81	-0.77	-0.94	-0.65	0.24	-1.15	-0.04	-0.86	-0.66	1.13	-0.44	-0.79
Reagent Validation 3	0.00	1.12	-0.36	-0.12	1.15	0.86	0.48	-0.98	1.10	1.15	-0.38	1.14	1.13
Matrix Spike Batch 1	0.81	-0.39	0.69	0.44	-0.83	-0.71	-0.04	1.54	0.03	-0.12	1.25	0.18	-0.21

Matrix Spike Batch 2	0.10	1.57	-0.99	-1.64	-0.61	-0.35	1.50	-1.24	-0.40	1.53	0.83	0.95	0.36
Matrix Spike Batch 3	0.78	-0.78	1.39	0.16	-0.74	-1.00	-0.87	0.11	-1.39	-1.02	-0.44	0.60	-1.63
Matrix Spike Batch 4	-1.65	-0.78	-0.59	-0.02	1.11	0.67	-0.93	-0.19	0.45	0.33	-0.49	-0.08	0.56
Matrix Spike Batch 5	-0.05	0.39	-0.50	1.05	1.08	1.39	0.35	-0.22	1.31	-0.72	-1.15	-1.64	0.92
Reagent Spike Batch 1	1.25	-0.86	-1.50	1.35	-0.35	-0.98	-0.66	-0.05	0.18	1.03	0.89	0.40	0.05
Reagent Spike Batch 2	0.11	1.60	0.66	-1.35	-0.70	-0.18	1.76	-0.24	0.68	1.03	1.25	0.26	0.31
Reagent Spike Batch 3	0.49	-0.57	-0.35	-0.23	-0.63	-0.78	-0.20	1.68	-1.73	-0.16	-0.45	1.31	-1.46
Reagent Spike Batch 4	-1.40	-0.52	1.10	-0.27	1.73	0.46	-0.38	-0.44	0.68	-1.05	-0.75	-0.73	-0.22
Reagent Spike Batch 5	-0.45	0.34	0.08	0.49	-0.04	1.48	-0.52	-0.95	0.18	-0.86	-0.93	-1.23	1.31
